# Antibody Stabilization of Peptide–MHC Multimers Reveals Functional T Cells Bearing Extremely Low-Affinity TCRs

**DOI:** 10.4049/jimmunol.1401785

**Published:** 2014-12-01

**Authors:** Katie Tungatt, Valentina Bianchi, Michael D. Crowther, Wendy E. Powell, Andrea J. Schauenburg, Andrew Trimby, Marco Donia, John J. Miles, Christopher J. Holland, David K. Cole, Andrew J. Godkin, Mark Peakman, Per Thor Straten, Inge Marie Svane, Andrew K. Sewell, Garry Dolton

**Affiliations:** * Institute of Infection and Immunity, Cardiff University School of Medicine, University Hospital, Cardiff CF14 4XN, Wales, United Kingdom;; † Center for Cancer Immune Therapy, Herlev University Hospital, DK-2730 Herlev, Denmark;; ‡ QIMR Berghofer Medical Research Institute, Brisbane, Queensland 4029, Australia; and; § Department of Immunobiology, King’s College London School of Medicine, Guy’s Hospital, London SE1 9RT, United Kingdom

## Abstract

Fluorochrome-conjugated peptide–MHC (pMHC) multimers are commonly used in combination with flow cytometry for direct ex vivo visualization and characterization of Ag-specific T cells, but these reagents can fail to stain cells when TCR affinity and/or TCR cell-surface density are low. pMHC multimer staining of tumor-specific, autoimmune, or MHC class II–restricted T cells can be particularly challenging, as these T cells tend to express relatively low-affinity TCRs. In this study, we attempted to improve staining using anti-fluorochrome unconjugated primary Abs followed by secondary staining with anti-Ab fluorochrome-conjugated Abs to amplify fluorescence intensity. Unexpectedly, we found that the simple addition of an anti-fluorochrome unconjugated Ab during staining resulted in considerably improved fluorescence intensity with both pMHC tetramers and dextramers and with PE-, allophycocyanin-, or FITC-based reagents. Importantly, when combined with protein kinase inhibitor treatment, Ab stabilization allowed pMHC tetramer staining of T cells even when the cognate TCR–pMHC affinity was extremely low (*K*_D_ >1 mM) and produced the best results that we have observed to date. We find that this inexpensive addition to pMHC multimer staining protocols also allows improved recovery of cells that have recently been exposed to Ag, improvements in the recovery of self-specific T cells from PBMCs or whole-blood samples, and the use of less reagent during staining. In summary, Ab stabilization of pMHC multimers during T cell staining extends the range of TCR affinities that can be detected, yields considerably enhanced staining intensities, and is compatible with using reduced amounts of these expensive reagents.

## Introduction

Fluorochrome-conjugated peptide–MHC (pMHC) multimers are now widely used in conjunction with flow cytometry for identifying Ag-specific T cell populations in direct ex vivo samples (1). The staining of T cells with multimerized pMHC circumvents the need for cellular activation required by other T cell detection methodologies and thereby allows detection of cells that fail to activate or that do not respond with the effector function(s) used for function-based profiling. pMHC multimer staining is also compatible with T cell phenotyping directly ex vivo by using a spectrum of fluorochrome-conjugated Abs specific for other T cell markers. Our previous studies have demonstrated that the binding affinity threshold for staining with pMHC class I (pMHC I) tetramers is significantly higher than that required for T cell activation (2). Thus, pMHC tetramers fail to stain all T cell subsets that are capable of responding to any given pMHC Ag. The disparity between the TCR affinity required for pMHC multimer staining and that required for T cell activation is highlighted when attempting to identify T cells specific for self-derived peptides (antitumor and autoimmune T cells), which generally bear TCRs that bind relatively weakly (*K*_D_ 10–300 μM) (3–5). This issue is further compounded when staining pMHC class II (pMHC II)-restricted T cells as, unlike the CD8 molecule, the CD4 coreceptor does not cooperate to aid TCR–pMHC binding (1, 6–12). The importance of this issue was highlighted by Sabatino and colleagues (13), who demonstrated that staining with pMHC II tetramers ex vivo underestimated the lymphocyte choriomeningitis virus glycoprotein_61–80_ and myelin oligodendrocyte glycoprotein_35–55_ CD4^+^ T cell populations by 4- and 8-fold, respectively. Demonstrations that pMHC tetramers can fail to detect the majority of responding cells in polyclonal antiviral and autoimmune T cell populations (13) highlight the pressing need to extend pMHC multimer technology to a point where it can be used to stain all T cells capable of responding to a given pMHC Ag (14, 15). Previously, we have described several improvements in pMHC multimer technology that extend the range of TCR–pMHC interactions that can be detected (1). The most promising of these technologies include use of anti-coreceptor Abs that enhance, rather than inhibit, staining (16, 17), use of protein kinase inhibitor (PKI) during staining (18), and use of ultra-bright high-valency reagents such as pMHC dextramers (15). Importantly, all of these methodologies can be used in combination for synergistic effects. In this study, we examined whether signal amplification via use of Abs to pMHC multimers could be used for improved detection. Our data revealed that simple addition of anti-multimer Ab during pMHC tetramer or dextramer staining can result in substantial improvements in staining intensity even when a log-fold lower concentration of reagent was used. We anticipate that this improved methodology will become widely adopted due to the large potential cost saving and a substantial extension to the range of TCR affinities that can be detected with pMHC multimers.

## Materials and Methods

### Cells

T cell clones/lines and tumor-infiltrating lymphocytes (TILs) were cultured in RPMI 1640 media supplemented with penicillin and streptomycin (P/S), l-glutamine, 10% FBS, 0.01 M HEPES buffer, nonessential amino acids, sodium pyruvate (Life Technologies, Paisley, U.K.), 25 ng/ml IL-15 (PeproTech, Rocky Hill, NJ) (T cell clones and TILs only), and either 20 or 200 IU/ml IL-2 (aldesleukin, brand name Proleukin; Prometheus, San Diego, CA), depending on the stage of culture. Tumor cells and surrogate pancreatic β cells (19) were cultured in RPMI 1640 media supplemented with P/S, l-glutamine, and 10% FBS (R10). Adherent cells were detached from tissue culture flasks by gently rinsing the cells with calcium and magnesium chloride–free Dulbecco's PBS (Life Technologies), followed by incubation with Dulbecco's PBS and 2 mM EDTA at 37°C, until the cells detached.

We made use of the following HLA-A*0201 (HLA-A2)–restricted CD8^+^ T cell clones: 1) ILA1, which is specific for the human telomerase reverse transcriptase (hTERT)–derived peptide ILAKFLHWL (residues 540–548) (20) as well as four altered peptide ligands (APL), referred to as 8E, 4L, 5Y, and 3G, which bind to the ILA-1 TCR with varying affinities (2, 21); 2) 1E6 and 3F2, which recognize the ALWGPDPAAA epitope from preproinsulin (PPI: residues 15–24) and originate from the same patient with type 1 diabetes (19); and 3) VB6G4.24, which recognizes the heteroclitic peptide E**L**AGIGILTV (heteroclitic residue in boldface) from Melan A (residues 26–35) and was derived from TILs of a patient with malignant melanoma [patient MM909.24 (22)]. We also made use of the HLA-DRB1*0101 (HLA-DR1)–restricted CD4^+^ clone DCD10, which recognizes the PKYVKQNTLKLAT epitope from influenza A hemagglutinin (residues 307–319) (23). T cell clones were routinely expanded by restimulation with allogeneic PBMCs and PHA as previously described (24), then cultured for at least 14 d before being used for staining, unless stated otherwise.

Fresh blood samples were obtained by venipuncture from volunteers (heparinized) or buffy coats (EDTA treated) from the Welsh Blood Service in accordance with the appropriate ethical approval. PBMCs were isolated by density centrifugation over an equal volume of Lymphoprep (Axis Shields, Oslo, Norway). PBMCs were either used immediately or from cryopreserved samples, with the latter being treated with 10–50 μg/ml DNase-I (Roche, Burgess Hill, U.K.) for at least 20 min after thawing at 37°C. We find that it is preferable to use fresh samples, as previously frozen samples can exhibit higher background levels of staining with some pMHC multimers. Cells were frozen in FBS with 10% DMSO using a controlled-rate freezing device (CoolCell; Biocision, Larkspur, CA) as per the manufacturer’s instructions and viable cell numbers enumerated by trypan blue exclusion. Spiked samples were created by mixing clonal T cells (10^4^) with defrosted PBMCs (10^6^), with the latter being cultured (24-well plates at a density of 3 to 4 × 10^6^/well in 2 ml R10) for 1 d prior to staining. The spiked PBMCs were minimally HLA matched for the restricting HLA of the spiking clone and treated as PBMC.

### pMHC multimer assembly

Soluble biotinylated pMHC I and pMHC II were produced as previously described (12, 25). Tetramers were assembled over five separate 20-min steps with the successive addition of streptavidin-allophycocyanin or –R-PE conjugates (Life Technologies) to monomeric pMHC at a molar streptavidin:pMHC ratio of 1:4. Dextramer (Immudex, Copenhagen, Denmark) PE, allophycocyanin, and FITC conjugates were assembled with monomeric pMHC as previously described (15). Protease inhibitors (set 1; Merck, London, U.K.) and PBS (tetramers) or dextramer buffer (15) were added to give a final pMHC multimer concentration of 0.1 μg/μl (with regards to the pMHC component), stored in the dark at 4°C, and used within 3 d of assembly. The same monomeric pMHC were used when tetramers and dextramers were assembled for use within the same experiment.

### PKI treatment

Cells were treated with the PKI dasatinib (Axon Medchem, Reston, VA) at a final concentration of 50 nM (18) for 30 min at 37°C and then stained with tetramer or dextramer without washing or prechilling to 4°C. It is important to note that PKI is unstable when stored at 4°C, so 1 mM DMSO aliquots of PKI were stored at −20°C. Then for each experiment, working aliquots of 100 nM were prepared in PBS.

### Primary and secondary Abs

Mouse anti-PE (clones PE001, BioLegend, London, U.K.; and eBioPE-DLF, eBioscience, San Diego, CA), -allophycocyanin (clones APC003, BioLegend; and eBioAPC-6A2, eBioscience), and -FITC (clone FIT-22; BioLegend) primary (1°) unconjugated mAbs were used at a concentration of 10 μg/ml (0.5 μg/test). Unless otherwise stated, the 1° Abs sourced from BioLegend were used throughout this study. The goat anti-mouse conjugated secondary (2°) Abs (multiple adsorbed PE-, allophycocyanin-, or FITC-conjugated Ig polyclonal; BD Biosciences, Oxford, U.K.) were used at 2 μg/ml (0.1 μg/test). The fluorochrome conjugated to the 2° Abs were matched to the fluorochrome used for the initial pMHC multimer staining. Both anti-fluorochrome and anti-Ab Abs were spun at maximum speed in a microcentrifuge for 1 min to remove any aggregates before staining cells. The optimal amounts of 1° and 2° Abs were established during this study using an Ab matrix on the 1E6 T cell clone. The matrix covered a range of 1° and 2° Ab concentrations (0.25–2 μg and 0.025–0.2 μg, respectively), tested individually and in combination. The concentration used for this study was based upon the highest signal (1° and 2° Abs in combination) to noise (2° alone) ratio of fluorescent intensity.

### Cell staining and flow cytometry

The desired number of cells, which was typically 0.5–1 × 10^5^ of a T cell clone and 1–3 × 10^6^ TILs, PBMCs, T cell line, or spiked samples, was transferred to flow cytometry tubes. Cells were washed with buffer (PBS with 2% FBS) before proceeding to PKI treatment or tetramer/dextramer staining as required. Tetramer concentrations ranged from 0.02 to 2.4 μg (0.4–48 μg/ml with respect to the monomeric pMHC concentration) per stain in 50 μl buffer, and typically 0.3 or 0.5 μg (6 or 10 μg/ml) was used unless stated otherwise. Dextramer was used at 0.3 μg (6 μg/ml) per stain. Following tetramer/dextramer addition, cells were placed on ice and in the dark for 30 min. All subsequent Ab staining of the cells was performed for 20 min on ice and in the dark. Post–pMHC multimer staining, the cells were washed in buffer and labeled with anti-fluorochrome unconjugated 1° Ab, followed by two washes with buffer before the anti-Ab conjugated 2° Ab was added. Cells were washed with buffer then PBS and the violet LIVE/DEAD Fixable Dead Cell Stain, Vivid (Life Technologies) added and placed in the dark at room temperature (RT) for 5 min, and then Abs against cell-surface markers were added directly without washing. Samples were prepared for flow cytometry by washing once in buffer and resuspended in PBS or 2% paraformaldehyde (PFA). For whole-blood samples, 0.1–0.125 ml heparanized blood was added to prealiquoted tetramer in flow cytometry tubes and incubated for 10 min at RT, with 0.375–0.5 ml blood being used per condition. A one-step staining approach was adopted in which the anti-fluorochrome 1° Ab was added directly to the tetramer staining for 15 min at 4°C, followed by a mixture of Abs against cell-surface markers and incubated for a further 15 min at 4°C. RBCs were lysed by incubating for 10 min at 37°C with 2.5 ml lysis buffer (155 mM NH_4_Cl, 10 mM KHCO_3_, and 0.01 mM EDTA [pH 7.2]) and then washed by the addition of 2 ml of PBS. Lysis was repeated where necessary and samples were combined for the same condition and run immediately on the flow cytometer or fixed with 2% PFA for 20 min on ice before two washes with PBS. A dead stain was not used for the whole-blood samples, although DNA binding reagents could easily be incorporated during the staining protocol and may give tetramer stains with less background. The following mAbs were used depending on each experiment: anti–CD8-PE and anti–CD8-allophycocyanin/PE-vio770 (clone BW135/80; Miltenyi Biotec, Bergisch Gladbach, Germany); anti–CD3-PerCP (clone BW264/56; Miltenyi Biotec); anti–CD19-Pacific blue (clone HIB19; BioLegend); and anti–CD14-Pacific blue (clone M5E2; BioLegend). Typically, PBMC, spiked, and whole-blood samples were gated on single, viable (not for whole blood), CD19^−^CD14^−^CD3^+^ lymphocytes and displayed in bivariate CD8 versus tetramer/dextramer plots. T cell clones were typically gated on single, viable, CD8^+^ or CD4^+^ lymphocytes displayed as histograms of tetramer fluorescence. Data were acquired on an FACSCanto II (BD Biosciences) and analyzed with FlowJo software (Tree Star, Ashland, OR).

### Intracellular cytokine staining assay

Cells were washed from culture medium and incubated in resting media (RPMI 1640 supplemented with P/S, l-glutamine, and 5% FBS) for 24 h prior to activation. Subsequently, cells were incubated at 37°C for 4 h, with and without (±) APCs, at a 1:1 ratio, in 2 ml resting media (24-well culture plate with a total cell density of 3–6 × 10^6^/ml) containing GolgiStop and GolgiPlug (both from BD Biosciences), according to the manufacturer’s instructions. Cells were then stained as above with cognate or irrelevant tetramer, 1° and 2° Ab(s), viability dye, and Abs against desired cell-surface markers. Cells were prepared for intracellular cytokine staining (ICS) by incubation with Cytofix/Cytoperm (BD Biosciences) according to the manufacturer’s instructions (including wash steps), before staining for 20 min on ice with mouse anti-human IFN-γ–allophycocyanin Ab (clone 45-15; Miltenyi Biotec). Cells were stored overnight (4°C in the dark) in 2% PFA before flow cytometry and data analysis.

### [^51^Cr] release cytotoxicity assay

Target cells were labeled for 1 h at 37°C with 30 μCi chromium (sodium chromate in normal saline; PerkinElmer, Waltham, MA) per 1 × 10^6^ cells, washed with R10, and allowed to leach for a further hour at 37°C in R10 to remove any excess chromium from the cells. After chromium labeling, target cells were washed and plated at 2000 cells/well in 96-well tissue culture plates. T cells were added to give the desired T cell to target cell ratio and a final volume of 150 μl R10. Target cells were also incubated alone or with 1% Triton X-100 detergent to give the spontaneous and total chromium released from the target cells, respectively. After 4 h of incubation, at 37°C and 5% CO_2_, the supernatants were harvested (10% of total volume), mixed with 150 μl Optipahse supermix scintillation mixture (PerkinElmer) 96-well polyethylene terephthalate plates (PerkinElmer), sealed, and the amount of released chromium measured indirectly on a 1450 Microbeta counter (PerkinElmer). The percentage of specific target cell lysis by T cells was calculated according to the following formula: (experimental release [with T cells and target cells] − spontaneous release from target cells)/(total release from target cells − spontaneous release from target cells) × 100.

### Tetramer decay assays

T cell clone (5 × 10^5^) was pretreated with PKI then stained with cognate tetramer ± an anti-fluorochrome unconjugated 1° Ab ± a conjugated 2° Ab. Cells were washed with staining buffer, supernatant aspirated, and incubated with 10 μg anti–HLA-A2 Ab (clone BB7.2, allophycocyanin conjugated; eBioscience) or diluted in 3 ml buffer and incubated at RT in the dark. PKI was present throughout some of the decay assays. Cells were sampled at the times indicated in the results section, washed with excess buffer, and fixed with 2% PFA.

## Results

### Addition of an anti-fluorochrome Ab substantially improves the staining and detection of T cells with tetramer

**FIGURE 1.**
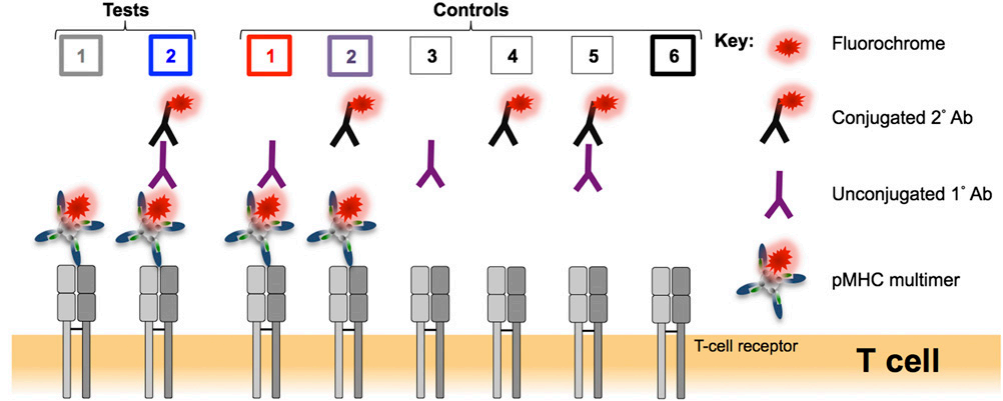
Schematic representation of the test and control conditions used in this study. Alongside a standard pMHC multimer (tetramer or dextramer) staining protocol (test 1), the binding of a mouse anti-fluorochrome unconjugated 1° Ab to the pMHC multimer associated fluorochrome followed by a goat anti-mouse conjugated 2° Ab (test 2) was tested to see whether the fluorescence intensity of pMHC multimer staining could be improved. A number of controls were performed: control 1: pMHC multimer with 1° Ab; control 2: pMHC multimer with 2° Ab; control 3: 1° Ab alone; control 4: 2° Ab alone; control 5: 1° and 2° Abs in combination; and control 6: unstained. The color coding for tests 1 + 2 and controls 1 + 2 + 6 is used throughout this study.

**FIGURE 2.**
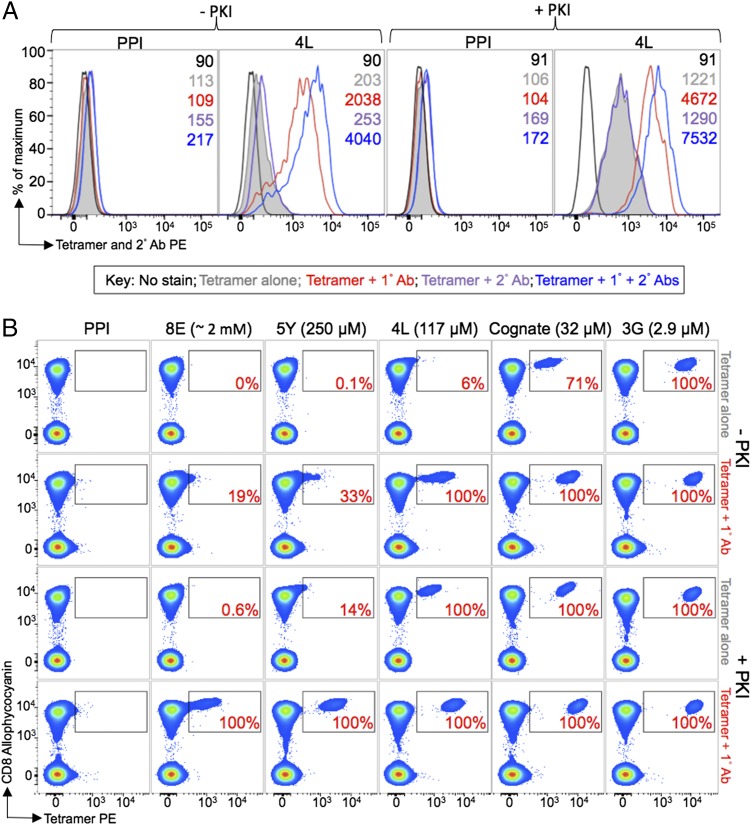
An anti-fluorochrome unconjugated Ab greatly enhanced the staining of T cells with tetramers when TCR–pMHC affinity is weak. (**A**) ILA1 hTERT-specific CD8^+^ T cells ± 50 nM PKI were stained with HLA-A2 PE-conjugated tetramers, assembled with the 4L peptide (*K*_D_ = 117 μM) or irrelevant (HLA-A2–ALWGPDPAAA, PPI) tetramers. Cells were stained with tetramers alone or with an anti-PE unconjugated 1° Ab, a 2° PE-conjugated Ab, or 1° + 2° Abs together. The MFI is shown for each histogram. (**B**) In a separate experiment, the ILA1 CD8^+^ clone was spiked in to PBMCs from an HLA-A2^+^ donor (used from frozen), treated ± PKI, and stained with PE-conjugated tetramers folded with cognate and APL agonists of the ILA1 clone (*K*_D_ [μM] shown in parentheses) or irrelevant epitope (as in A). Tetramers were used alone or in combination with anti-PE unconjugated 1° Ab. 2° Ab was not used in this experiment to highlight the unexpected effect of 1° anti-fluorochrome Ab. The percentage recovery of gated cells is displayed in the *inset* and was determined relative to the proportion of cells that stained with the 3G variant (considered 100%) after subtracting any background seen with the PPI tetramer. Display is based on viable CD3^+^CD14^−^CD19^−^ cells.

We have previously described an important disparity between the TCR–pMHC affinity required for T cell activation and that required for effective capture of pMHC tetramers from solution (2). This difference means that pMHC tetramers do not stain all Ag-specific T cell populations (2) and represents a particular problem when pMHC multimers are used to stain self-specific or pMHC II–restricted T cells with weaker affinity TCRs (1, 3–5, 13). We made use of the ILA1 T cell clone that recognizes the HLA-A2–restricted hTERT-derived peptide ILAKFLHWL. This hTERT peptide is not naturally presented at the tumor cell surface (20) and therefore provides a model system that is uncomplicated by the possibility of a natural ligand. We have previously characterized a wide range of APL that act as agonists of the ILA1 T cell and that range in affinity for the ILA1 TCR from *K*_D_ ∼3 μM to K_D_ ∼2 mM by surface plasmon resonance while binding to HLA-A2 equally well (2, 21). The ILA1 T cell system therefore enables the TCR–pMHC affinity to be varied, whereas other variables such as surface density of TCR and CD8 remain identical. Two of the weaker APL in this system, 4L and 5Y, bind with a *K*_D_ of 117 and ∼250 μM, respectively, and provide a good model for weakly binding autoimmune TCRs. A further APL, 8E, still acts as a good agonist of ILA1 T cells when supplied exogenously at a concentration of 1 μM (2, 21) yet binds to the TCR with a *K*_D_ ∼2 mM by extrapolation of response units from surface plasmon resonance experiments. Previously, we have devised a number of novel techniques that lower the detection limit of pMHC multimer staining. These include CD8-enhanced tetramers (10) and the use of a PKI to prevent the internalization of TCRs from the cell surface that have not productively captured pMHC multimer from solution (18). The use of PKI considerably enhanced the range of TCR affinities amenable to detection with pMHC tetramers (18). When pMHC multimers are used in conjunction with PKI, the multimers remain at the cell surface (15, 18). We reasoned that, in the presence of PKI, pMHC multimers would be available for further signal amplification using fluorochrome-conjugated Abs. We therefore set up a staining protocol as shown in Fig. 1 that included combinations of an anti-PE unconjugated 1° and anti-Ab PE-conjugated 2° Abs as indicated. Initial experiments were conducted using the weak ILA1 ligand HLA-A2–ILA**L**FLHWL (4L; *K*_D_ = 117 μM). Tetramers of the weak 4L ligand barely stained the ILA1 T cell clone in the absence of 50 nM PKI (Fig. 2A). Addition of PKI enhanced staining by >6-fold. Further inclusion of 1° and 2° Abs enhanced staining by ∼20-fold in the absence of PKI and by ∼6-fold in the presence of PKI (Fig. 2A). These results show that inclusion of Ab stabilization can have marked effects on staining even when PKI is not included to preclude TCR internalization. The additional increase in mean fluorescence intensity (MFI) observed using anti-pMHC multimer Ab in the presence of PKI confirmed that these two techniques could be used in combination. Moreover, there was a 10-fold enhancement in staining with tetramer and 1° Ab compared with staining with tetramer alone (conditions: control 1 and test 1 in Fig. 1, respectively). pMHC tetramer staining in the presence of the 1° Ab was also almost four times brighter in the presence of PKI. This substantial increase in pMHC tetramer staining in the presence of a 1° Ab, but in the absence of any further fluorochrome provided by a 2° Ab staining, was highly unexpected. We next studied this unanticipated large increase in MFI afforded by simple addition of anti-fluorochrome Ab during pMHC tetramer staining by examining recovery of the ILA1 clone spiked into an HLA-A2^+^ PBMC sample using tetrameric forms of a number of different APL (Fig. 2B). Complete recovery of spiked ILA1 T cells was achieved in all cases when the 3G ligand (*K*_D_ ∼3 μM) was used (considered as 100% recovery). Only 71% of the cells were recovered with pMHC tetramers of the cognate, hTERT-derived HLA-A2–ILAKFLHWL ligand (*K*_D_ ∼35 μM) in the absence of PKI treatment. This was increased to full recovery when either PKI or 1° Ab were included. The greatest fluorescence intensity was seen when both PKI and 1° Ab were included. The effects of including 1° Ab during pMHC tetramer staining became even more exaggerated with the 4L ligand (*K*_D_ = 117 μM) in which recovery with normal tetramer staining in the absence of PKI treatment or Ab stabilization was extremely poor (6%). With the 5Y ligand (*K*_D_ ∼250 μM), full recovery was *only* achieved when tetramer was used with PKI and 1° Ab in combination (Fig. 2B). Remarkably, full recovery of ILA1 clone was still possible when tetramers of the 8E ligand (*K*_D_ ∼2 mM) were used in conjunction with PKI and 1° Ab. In the past, we have failed to recover cells using the 8E ligand using even our best technology to date of PKI treatment in conjunction with higher valency, ultra-bright, pMHC dextramers (15). Thus, the simple technology described in this study extends the range of TCR–pMHC interactions that are amenable to detection using pMHC multimers beyond the current limit possible for these reagents.

### Anti-fluorochrome Abs alone or in combination with conjugated secondary Abs substantially improve staining of autoimmune T cells with pMHC tetramers

**FIGURE 3.**
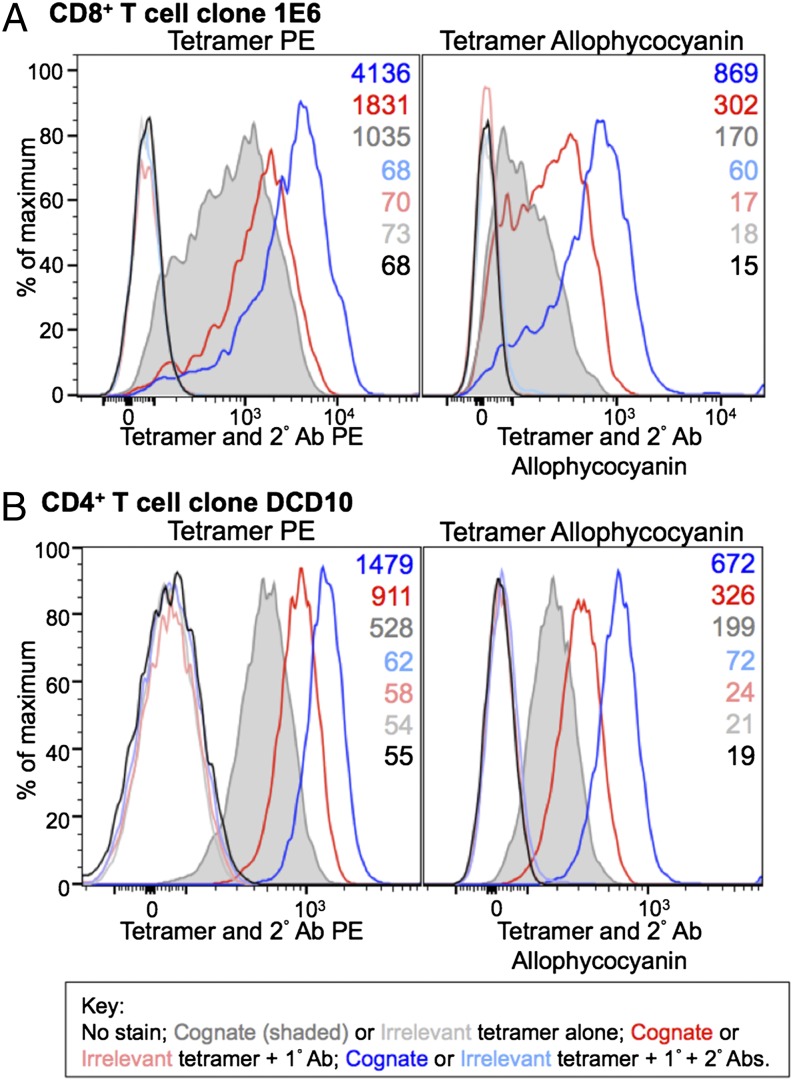
Enhanced tetramer staining of an autoimmune T cell and an MHC II–restricted T cell with anti-fluorochrome unconjugated and secondary conjugated Abs. (**A**) PKI-treated CD8^+^ T cell clone 1E6 was left unstained or stained with PE and allophycocyanin-conjugated cognate HLA-A2–ALWGPDPAAA (PPI) cognate and HLA-A2–ELAGIGILTV (Melan-A) irrelevant tetramers, alone or with an anti-fluorochrome unconjugated 1° Ab ± PE- or allophycocyanin-conjugated 2° Ab. (**B**) The CD4^+^ T cell clone, DCD10, was left unstained or stained from culture with cognate HLA-DR1–PKYVKQNTLKLAT (hemagglutinin of influenza) or irrelevant HLA-DR1–DRFYKTLRAEQASQ (p24 Gag of HIV) PE- and allophycocyanin-conjugated tetramer as described in (A). MFIs are shown at the *right* of each graph.

We next looked at whether the increase in the MFI of staining with pMHC tetramers observed with the ILA1 model system was applicable with other T cells and with pMHC multimers conjugated to other fluorochrome molecules. For these experiments, we used the 1E6 T cell clone that exhibits glucose-dependent killing of HLA-A2^+^ human pancreatic β-cells and was derived from a patient with type 1 diabetes (19). 1E6-mediated killing occurs via the PPI-derived peptide ALWGPDPAAA presented by the disease risk allele HLA-A2 (19). The 1E6 TCR binds to its cognate HLA-A2–ALWGPDPAAA with a *K*_D_ of >250 μM (26, 27). Fig. 3A shows results with both PE and allophycocyanin reagents using anti-fluorochrome unconjugated 1° Ab clones PE001 and APC003, respectively. Inclusion of a 1° Ab and further fluorescence enhancement with anti-Ab conjugated 2° Ab increased the MFI of staining by ∼4-fold and >5-fold for the PE and allophycocyanin staining, respectively. In both cases, and as seen in the ILA1 system (Fig. 2A), the majority of this increase in fluorescence intensity was apparent in the absence of a 2° Ab. Thus, inclusion of a 1° Ab during pMHC tetramer staining can substantially increase the intensity of staining of an autoimmune T cell clone with pMHC tetramer. We also tested another anti-PE 1° Ab (eBioPE-DL; BioLegend) and an anti-allophycocyanin 1° Ab (eBioAPC-6A2; BioLegend), which gave increases of 3.5- and 2.4-fold, respectively in the absence of a 2° Ab (data not shown). Similar levels of enhancement were also observed with FITC-conjugated reagents (dextramer FITC with corresponding reagents, data not shown), showing that the substantial benefits afforded by addition of anti-fluorochrome and anti-Ab Abs when staining cognate autoimmune T cells are generally applicable and evident regardless of which fluorochrome is used.

### Anti-fluorochrome Abs alone or in combination with conjugated secondary Abs enhance staining of CD4 T cells with pMHC II tetramers

The weaker average affinity of TCRs derived from MHC II–restricted T cells (3) and lack of coreceptor help from CD4 (1) means that it is generally more difficult to stain cognate T cells with pMHC II tetramer than pMHC I tetramers (28), and pMHC II tetramers have been shown to miss the majority of Ag-specific T cells in polyclonal antiviral and autoimmune populations (13). Given this limit in visualization, we next examined whether inclusion of anti-fluorochrome and anti-Ab Abs could be beneficial in the pMHC II tetramer setting. For these experiments, we made use of the HLA-DR1–restricted, influenza-specific T cell clone DCD10. This antiviral T cell clone stains reasonably well with cognate tetramer, with MFIs of 528 and 199 for the PE and allophycocyanin reagents, respectively (Fig. 3B). Addition of an anti-PE or -allophycocyanin unconjugated 1° Ab, used alone or in combination with an anti-Ab conjugated 2° Ab enhanced the staining of this T cell clone by 1.7- and 2.8-fold for PE reagents and 1.6- and 3.3-fold for allophycocyanin reagents, respectively. Thus, stabilization of pMHC II tetramers can improve the intensity of cell staining with these reagents.

### Ab stabilization illuminates low-affinity T cells otherwise undetected by conventional tetramer staining and with lower concentrations of tetramer

**FIGURE 4.**
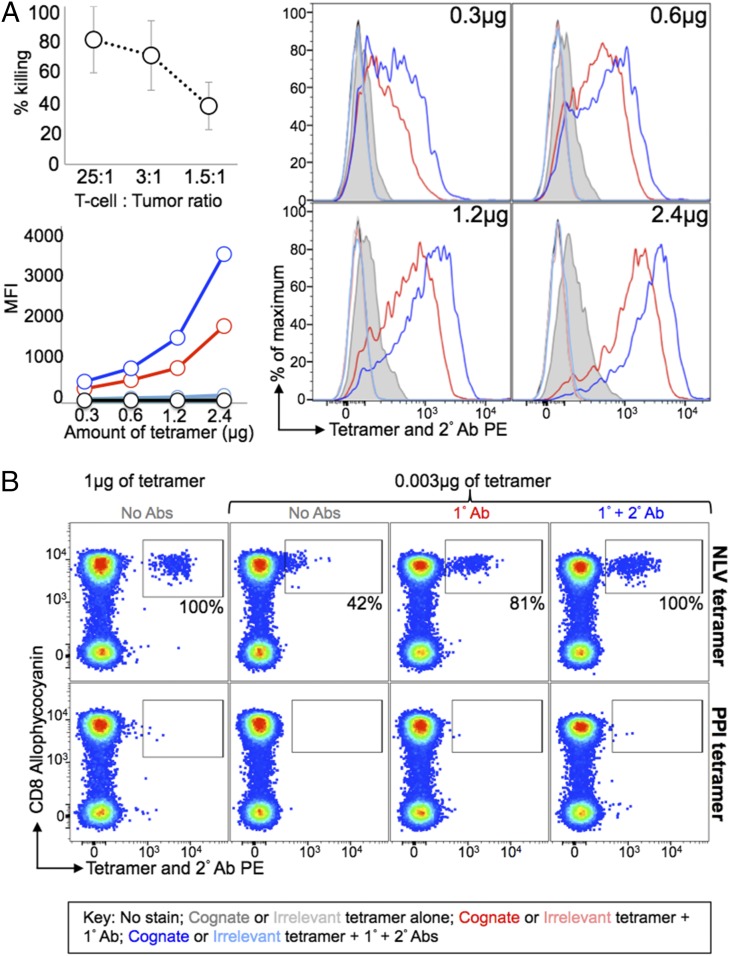
Anti-fluorochrome and secondary Abs enable staining of weak-avidity T cells at lower concentrations of tetramer. (**A**) The CD8^+^ VB6G4.24 T cell clone, grown from TILs from a malignant melanoma patient, kills autologous tumor (*top left panel*). The clone was stained with various amounts of PE-conjugated cognate HLA-A2–ELAGIGILTV (Melan-A) and irrelevant HLA-A2–ALWGPDPAAA (PPI) tetramers. Fresh cells were left unstained or stained with tetramer alone or with an anti-PE unconjugated 1° Ab ± PE conjugated 2° Ab. The *bottom left panel* shows the MFI of tetramer staining, which is shown in the histograms (*right panel*). (**B**) Fresh HLA-A2^+^ PBMC was stained with HLA-A2–NLVPMVTAV (pp65 of CMV, *top panel*) or PPI (*bottom panel*) PE-conjugated tetramers. Cells stained with 0.003 μg were either stained with tetramer alone or tetramer with a combination of 1° and 2° Abs, as described in (A). The proportion of cells that stained with 0.003 μg of tetramer is expressed as a percentage (*inset*) of the cells that stained with 1 μg of tetramer after subtraction of any background seen with the PPI tetramer (*bottom panel*). PBMC were stained for viability and Abs against CD8, CD3, CD14, and CD19. No pretreatment with PKI was used throughout.

We next examined the effect of 1° and 2° Abs on pMHC tetramer staining of the tumor-specific CTL clone VB6G4.24 that was grown from the TILs derived from a patient with stage IV malignant melanoma (22). This clone efficiently kills the patient's autologous tumor even at low E:T ratios but does not stain by conventional pMHC tetramer staining even when high amounts of reagent were used (Fig. 4A). Tetramer staining of this clone was negligible even with 2.4 μg of tetramer (with respect to the pMHC component). Addition of an anti-PE unconjugated 1° Ab enabled staining of this clone with most of the cognate pMHC tetramer amounts tested and as low as 0.6 μg (with respect to the pMHC I component) of tetramer. Further inclusion of an anti-Ab PE-conjugated 2° Ab doubled the staining observed with the 1° Ab, but as before, the majority of the enhancement in MFI was provided by inclusion of the 1° Ab alone.

Tetramers are most commonly used to stain antipathogen CD8^+^ T cells and have excelled for such applications (1, 29, 30). The TCRs of CD8^+^ T cells raised against non–self-peptides tend to bind with relatively strong affinity to their cognate pMHC Ag (*K*_D_ 0.1–10 μM) (3, 5, 31). We used tetramers to stain CMV-specific T cell populations directly ex vivo and showed that inclusion of Ab allowed full recovery of CMV-specific T cells from PMBC samples even when >300-fold lower concentrations of pMHC tetramer were used (just 3 ng with respect to pMHC). CMV-specific T cells could not be detected as a separate distinct population of cells in the absence of Ab when this amount of pMHC tetramer was used for staining (Fig. 4B). Thus, the methodology described in this study allows recovery of T cells with dramatically lower amounts of pMHC multimer regardless of the TCR–pMHC affinity and is compatible with ex vivo staining of PBMC. Lower concentrations of tetramer could also be used when recovering the 1E6 PPI-specific clone from spiked HLA-A2^+^ PBMC samples. Addition of a 1° Ab resulted in recovery of >80% of the 1E6 cells even when 25-fold less pMHC tetramer was used. Inclusion of a 2° Ab allowed full recovery of cells, even when 25-fold less tetramer was used (data not shown).

### Ab stabilization of pMHC tetramer and dextramers gives superior recovery of T cells from multiple sources

**FIGURE 5.**
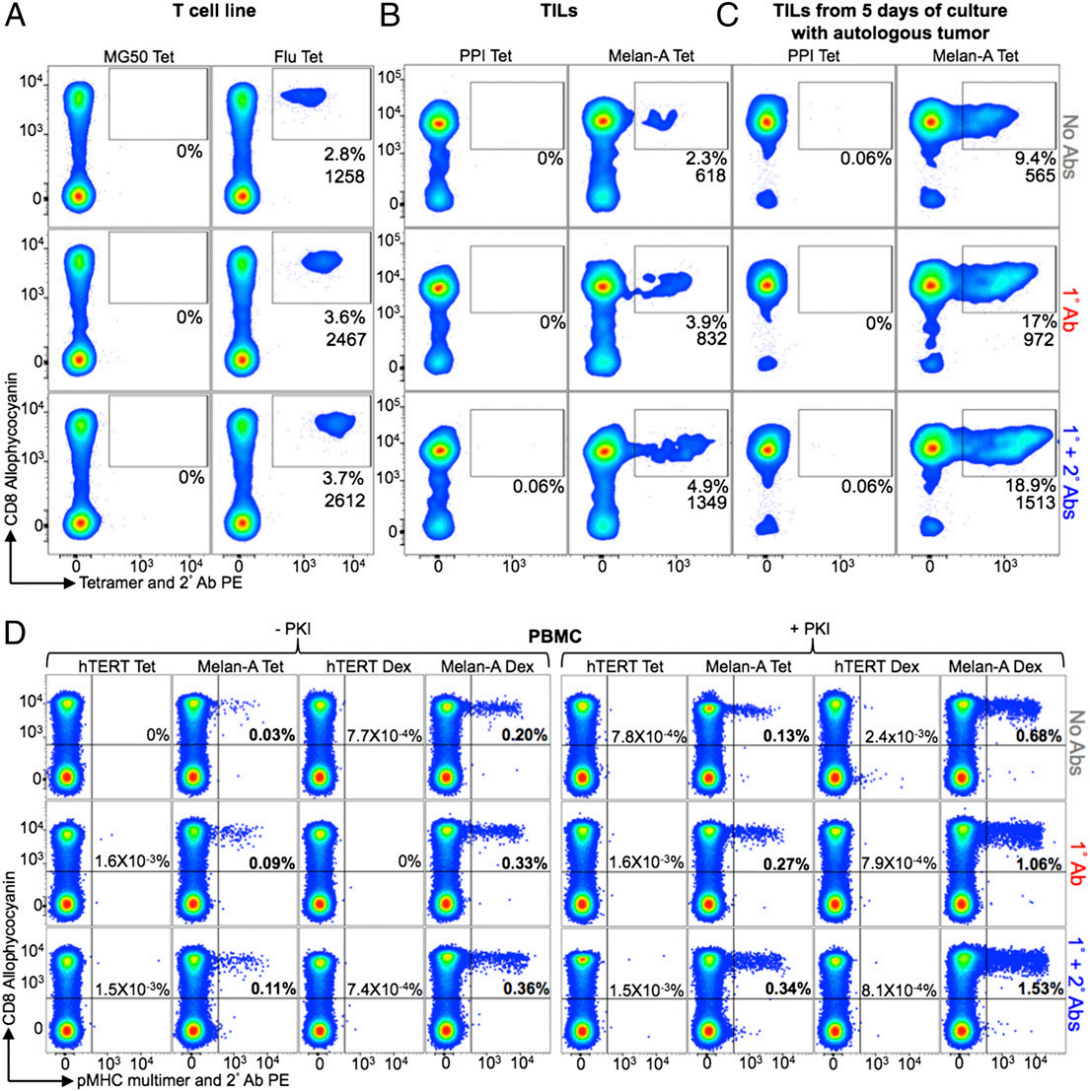
Ex vivo staining and detection of T cells is improved by the addition of an anti-fluorochrome and conjugated secondary Ab to standard pMHC multimer staining protocols. (**A**) A T cell line primed with GILGFVFTL peptide from the influenza virus (flu) was treated with PKI and stained with cognate HLA-A2 PE-conjugated cognate and control (HLA-A2-RLGPTLMCL from MG50 protein) tetramers (Tet), alone or in combination with anti-PE unconjugated 1° Ab ± a PE-conjugated 2° Ab. (**B**) TILs from an HLA-A2^+^ metastatic melanoma patient were treated with PKI and stained with HLA-A2–ELAGIGILTV (Melan-A) or HLA-A2–ALWGPDPAAA (PPI) PE-conjugated tetramers and Abs as in (A). (**C**) The staining described in (B) was performed on TILs that had been cultured with autologous tumor for 5 d. (**D**) HLA-A2^+^ PBMCs taken directly ex vivo were incubated ± PKI and stained with HLA-A2–ELAGIGILTV or HLA-A2–ILAKFLHWL (hTERT) PE-conjugated tetramers or dextramers (Dex) and Abs as described in (A). Samples were minimally stained for viability, CD3, and CD8, with CD14 and CD19 also being stained in (C). The tetramer^+^ cells are expressed as a percentage of total cells (A and B) or CD8^+^ cells (C) and the MFIs are shown (*inset*).

We next compared pMHC multimer staining of a T cell line, TILs, and PBMC samples using the following conditions: 1) pMHC multimer alone (test 1, Fig. 1); 2) pMHC multimer + anti-PE unconjugated 1° Ab (control 1, Fig. 1); and 3) pMHC multimer + the 1° Ab + anti-Ab PE-conjugated 2° Ab (test 2, Fig. 1) (Fig. 5). Fig. 5A shows classic tetramer staining of an HLA-A2–restricted influenza matrix-specific T cell line. As expected, the cognate CD8^+^ T cells in this antiviral line stain well with tetramer. Nevertheless, inclusion of a 1° Ab during staining almost doubled the MFI and resulted in recovery of a ∼25% greater population of cells. Further inclusion of a 2° Ab resulted in a further minor increase in both MFI and percent population recovered. We next applied the same conditions in the presence of PKI for staining of HLA-A2–ELAGIGILTV-specific cells in TILs expanded from a melanoma lesion (Fig. 5B). A total of 2.3% of the cells in these TILs stained with Melan-A–specific pMHC tetramer. The size of this population almost doubled when 1° Ab was included in the protocol. The population recovered increased from 3.9 to 4.9% when a 2° Ab was also included. In an independent assay using the same TILs, the Melan-A specific T cell population segregated into two clean populations when 1° and 2° Abs were included with tetramer (Supplemental Fig. 1A). The VB6G4.24 T cell clone shown in Fig. 4A was cloned from these TILs and is effective at killing patient autologous tumors. This clone does not stain with pMHC tetramer (Fig. 4A), so we assume that this clone is one of the T cell clonotypes that fails to stain using tetramer alone in the presence of PKI in Fig. 5B. Importantly, staining can be recovered when 1° Ab and 1° + 2° Abs were included in the staining protocol. Enhanced tetramer staining was also seen when tumor-specific T cells were relatively abundant. The aforementioned TILs were enriched for Melan-A–specific cells by coculture with autologous tumor for 5 d. Twice as many cells were stained with Melan-A tetramers when 1° and 2° Abs (9.4% versus 18.9%) were included, which represents a considerable increase in the number of T cells being detected (Fig. 5C). Thus, pMHC tetramer staining in the absence of Ab stabilization can fail to recover effective cognate CD8^+^ T cells resulting in a large underestimation of the size of an Ag-specific T cell population. This large underestimation of effective, Ag-specific CD8^+^ T cells with pMHC I tetramer is in accordance with a previous study that showed that most Ag-specific CD4^+^ T cells could not be detected by pMHC II tetramer staining of ex vivo samples (13).

pMHC multimers are most commonly used for detecting Ag-specific T cell populations directly ex vivo. To compare various methodologies available in this context, we took advantage of the fact that there is a relatively large population of naive T cells in HLA-A2^+^ individuals that recognize a commonly used variant of a self-peptide from the Melan-A protein (sequence ELAGIGILTV) due to a hardwired germline-encoded recognition motif (32, 33). Some of these self-specific CD8^+^ T cells can be detected by regular tetramer staining (Fig. 5D). The size of this population increases from 0.03% of CD3^+^CD8^+^ cells to 0.09 and 0.11% of cells when 1° Ab and 1° + 2° Abs were included, respectively. We recently used this system to show that pMHC dextramers were better at recovering low-avidity T cells when compared with pMHC tetramers, with the best recoveries seen when dextramers were used in the presence of PKI (15). The same effect was also observed in this study in which use of pMHC dextramer gave 6.6-fold more cells being recovered than with the equivalent pMHC tetramer alone in the absence of PKI and 5.2-fold in the presence of PKI (Fig. 5D). We also tested the effect of Ab with pMHC dextramers, which increased recovery from 0.20% for dextramer alone, to 0.33 and 0.36% when 1° Ab and 1° + 2° Abs were included, respectively. In the presence of PKI, the recovery of cells increased from 0.68% (dextramer alone) to 1.06 and 1.53% when 1° Ab and 1° + 2° Abs were included. In accordance with our earlier results, higher numbers of CD8^+^ T cells stained with pMHC dextramer than with pMHC tetramer, reflecting the ability of these higher valency reagents to stain T cells bearing lower-affinity TCRs. Optimal recovery was seen with PKI + pMHC dextramer + 1° + 2° Abs. The population recovered using this combination was 50-fold greater than could be recovered with pMHC tetramer alone, with no PKI, and 11-fold greater when pMHC tetramer was used with PKI. Staining of PKI-treated PBMCs from a second donor with Melan-A dextramers + 1° + 2° Abs recovered 11-fold more cells than Melan-A tetramers alone (Supplemental Fig. 1B). Overall, in terms of cellular recovery and regardless of PKI treatment, pMHC dextramer + 1° + 2° Ab > pMHC dextramer + 1° Ab > pMHC dextramer > pMHC tetramer + 1° + 2° Abs > pMHC tetramer + 1° Ab > pMHC tetramer.

### Ab stabilization is compatible with whole-blood staining with pMHC tetramers

**FIGURE 6.**
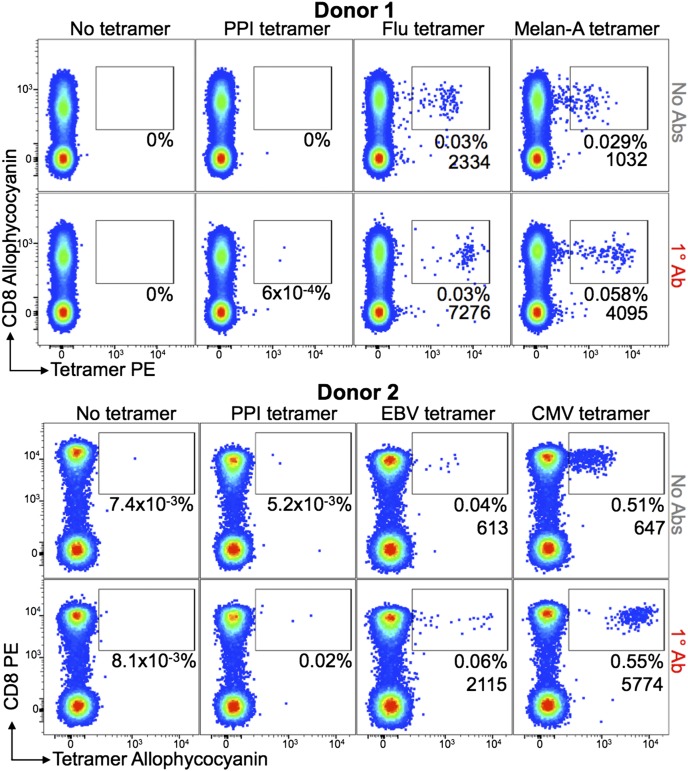
Ab stabilization is compatible with whole-blood staining with pMHC multimers. Fresh heparinized whole blood from two HLA-A2^+^ donors was treated with PKI then added to prealiquoted PE- (donor 1) or allophycocyanin-conjugated (donor 2) tetramers. Both donors were stained with HLA-A2–ALWGPDPAAA (PPI) tetramer. Donor 1 was stained with HLA-A2–GILGFVFTL (influenza [Flu]) and HLA-A2–ELAGIGILTV (Melan-A) tetramers and donor 2 with HLA-A2–CLGGLLTMV (EBV) and HLA-A2–NLVPMVTAV (CMV) tetramers. Anti-PE or allophycocyanin unconjugated 1° Ab was added directly to the cells, followed by a mixture of Abs against cell-surface markers (CD19, CD14, CD3, and CD8) before lysis of RBCs. A total number of 3 × 10^5^ and 1 × 10^5^ CD3^+^CD19^−^CD14^−^ cells were acquired from 0.5 ml and 0.375 ml of whole blood for donors 1 and 2, respectively. The percentage of cells residing within the gate and the MFI of this population are shown for each plot.

We next tested the compatibility of Ab stabilization when staining whole blood with pMHC tetramers. Blood samples from two HLA-A2^+^ healthy donors were stained with four different pMHC tetramers as described in the *Materials and Methods*. Donor 1 had populations of CD8^+^ T cells that stained with HLA-A2-GILGFVFTL (influenza) and HLA-A2–ELAGIGILTV (Melan-A) tetramers with MFIs of 2334 and 1032, respectively, for the gates shown in Fig. 6. These MFIs increased to 7276 and 4095 when a 1° anti-PE Ab was included, and the number of cells staining with the ELAGIGILTV Melan-A–specific reagent doubled. Donor 2 had populations of T cells that stained with HLA-A2–CLGGLLTMV (EBV) and HLA-A2-NLVPMVTAV (CMV) tetramers, with the 1° Ab increasing the MFI of tetramer staining from 613 to 2115 and 674 to 5774, respectively. We conclude that Ab stabilization of pMHC multimers is compatible with whole-blood staining protocols.

### Ab stabilization of pMHC tetramers improves recovery of T cells that have recently been exposed to Ag

**FIGURE 7.**
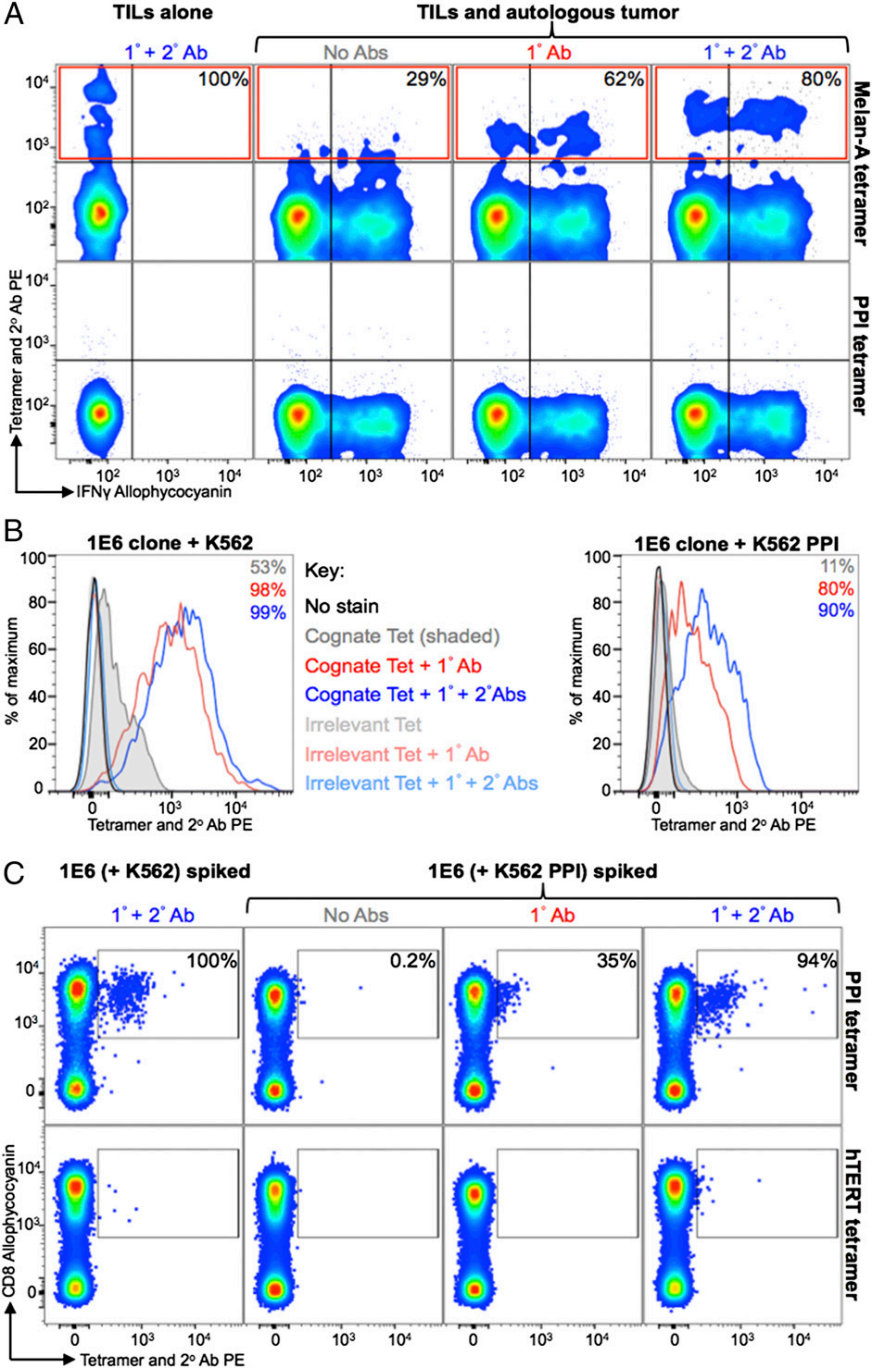
Activated T cells can be detected when tetramers were used with anti-fluorochrome and secondary Abs. (**A**) TILs from a HLA-A2^+^ metastatic melanoma patient were incubated with brefeldin A and monensin, ± autologous tumor. Cells were stained with cognate HLA-A2–ELAGIGILTV (Melan A) or HLA-A2–ALWGPDPAAA, PPI) PE-conjugated tetramers (Tet) alone or further labeled with an anti-PE unconjugated 1° Ab ± PE conjugated 2° Ab. Cells were also stained intracellularly for IFN-γ. Tetramer^+^ cells (red box) from the TILs with autologous tumor are expressed as a percentage (*inset*, *top panel*) of tetramer^+^ cells (Tet + 1° + 2° Abs) from the TILs alone after subtracting the number of gated cells seen with the PPI tetramer (*bottom panel*). (**B**) The CD8^+^ T cell clone 1E6, specific for ALWGPDPAAA from PPI, was incubated with K562-A2^+^ or K562-A2^+^ that express PPI (the latter process and present the cognate epitope). At 24 h postincubation, the cells were treated with 50 nM PKI and stained with cognate or irrelevant (HLA-A2–ILAKFLHWL, hTERT) tetramer alone or further labeled with an anti-PE 1° Ab ± a PE-conjugated 2° Ab. The percentages of 1E6 detected above the staining with an irrelevant tetramer are displayed for each histogram. (**C**) From the same experiment in (B), the 1E6 clone that had been cocultured with K562 or K562-PPI was spiked in to CD3/CD28-amplified PBMCs (HLA-A2^+^) and then PKI treated and stained as described in (B). The proportion of HLA-A2–ALWGPDPAAA tetramer^+^ cells (gated) from 1E6 activated with K562-PPI is expressed as a percentage of HLA-A2–ALWGPDPAAA tetramer^+^ cells (stained with Tet + 1° + 2° Abs) from 1E6 cultured with K562 (*top panel*). The gates are based on staining with an irrelevant tetramer (*bottom panel*). All cells were stained for viability and expression of CD3 and CD8.

Ag engagement is known to trigger and downregulate TCR from the T cell surface (34) and makes pMHC multimer staining more difficult due to low TCR density (15). This aspect could be problematic if staining pathogen-specific T cells during acute or chronic infections. It is also likely that self-specific T cells, be they antitumor T cells in TILs or of autoimmune origins, will have recently encountered their cognate Ag in vivo prior to staining. We mimicked this situation by exposing TIL to autologous tumor or autoimmune T cells to target cells endogenously producing cognate self-Ag prior to examining cellular recovery with pMHC multimers. TILs from an HLA-A2^+^ metastatic melanoma patient were incubated with brefeldin A and monensin ± autologous tumor for 4 h prior to staining with Melan A tetramer and intracellularly with anti–IFN-γ Ab (Fig. 7A). After exposure to tumor, tetramer alone, tetramer with an anti-PE unconjugated 1° Ab ± anti-Ab PE-conjugated 2° Ab recovered 29, 62, and 80% of the ELAGIGILTV-specific T cells that could be recovered without prior exposure to tumor, respectively. We also examined staining of the 1E6 PPI-specific T cell clone after incubation with K526 cells expressing HLA-A2 or K526 cells expressing HLA-A2 and PPI, with the latter termed "surrogate pancreatic β cells." Staining of 1E6 cells was very poor with tetramer alone postexposure to K526 cells expressing the cognate Ag compared with when 1° Ab ± 2° Ab, were used (11, 80, and 90% of the cells stained with each condition, respectively; Fig. 7B). This staining pattern was reflected when 1E6 cells that had been incubated with K526 cells ± cognate Ag were spiked into HLA-A2^+^ PBMC. Tetramer + 1° + 2° Abs was able to recover 94% of the cells that had been exposed to K526 surrogate β cells, whereas recovery with tetramer + 1° Ab or tetramer alone was 35 and 0.2%, respectively (Fig. 7C). These results confirm our previous findings that tetramers are poor at recovering T cells following exposure to cognate Ag (15) but show that the inclusion of 1° or 1° + 2° Abs against tetramer as described in Fig. 1 can reverse most of this effect and enable effective T cell staining (Fig. 7B, 7C).

### Ab stabilizes pMHC tetramer at the T cell surface

**FIGURE 8.**
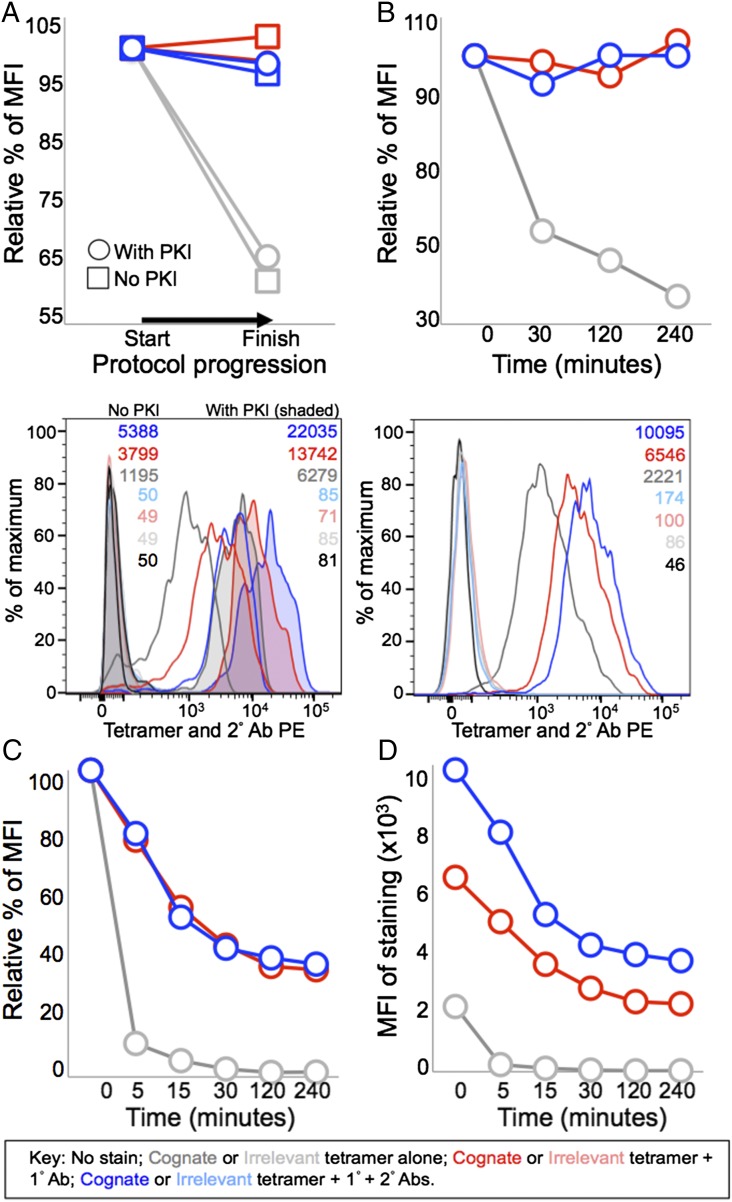
Stabilization with an anti-fluorochrome Ab preserves tetramer staining at the surface of T cells. (**A**) The CD8^+^ T cell clone 3F2 was treated with 50 nM PKI, or left untreated, and stained with cognate HLA-A2–ALWGPDPAAA (from preproinsulin) PE-conjugated tetramers or irrelevant HLA-A2–NLVPMVTAV (from CMV) tetramers. Cells were stained with tetramer alone (gray) or tetramer with anti-PE unconjugated 1° Ab (red) ± a PE-conjugated secondary 2° Ab (blue). Once stained with tetramer ± 1° Ab ± 2° Ab (Start), the cells were taken through three incubations (20 min on ice) and associated wash steps (two times) before being analyzed (Finish). The histogram shows the staining at the start of the assay. (**B**) 3F2 was treated with PKI and stained as in (A), then diluted in an excess volume of buffer (3 ml), and incubated at RT for the times shown. The histogram shows the staining at the start of the assay. (**C** and **D**) From the same experiment in (B), cells were incubated at RT with an anti–HLA-A2 Ab (BB7.2) in 0.1 ml of buffer and samples taken at the times shown. Graphs display the percentage of tetramer staining relative to the start of the experiment for each condition (A–C) or the MFI (C). PKI was present throughout the assay for (B)–(D).

When we initially saw that inclusion of an unconjugated anti-fluorochrome Ab dramatically improved the MFI of staining during pMHC tetramer staining, we considered the possibility that the 1° Ab might function by somehow stabilizing the fluorochrome and/or enhancing its ability to emit detectable fluorescence. Subsequent experiments showed the same affect with different Ab clones and MFI enhancements with anti-allophycocyanin or anti-FITC Abs and appropriate fluorochrome-conjugated pMHC multimers (Fig. 3 for allophycocyanin data, FITC not shown). We further tested the stabilization of fluorochrome hypothesis by using an unconjugated 1° Ab against the streptavidin component of the tetramer. Anti-streptavidin 1° Ab enhanced the MFI of tetramer staining, although not as impressively as the anti-fluorochrome Ab tested alongside (data not shown). The reduced effect of an anti-streptavidin Ab compared with anti-PE Ab may reflect steric difficulties in Ab binding to streptavidin in a PE-conjugated pMHC tetramer. Overall, there was fluorochrome-independent 1° Ab-induced enhancement of tetramer staining regardless of which anti-pMHC tetramer Ab was used. This made it more likely that the Ab was functioning by stabilizing pMHC multimer at the T cell surface during the staining protocol. We formally tested this hypothesis using the PPI-specific 3F2 CD8^+^ T cell clone that bears an identical TCR to the 1E6 T cell clone. T cells were stained ± PKI with cognate and control pMHC tetramer. Samples were fixed with PFA immediately after staining and washed or taken through subsequent incubations and washing steps. Surprisingly, almost half of the staining with pMHC tetramer was lost (Fig. 8A); this loss was greatest in the absence of PKI. In contrast, the intensity of the initial staining was maintained in the presence of PKI and 1° Ab (Fig. 8). Tetramer staining was completely stable when 1° Ab or 1° + 2° Ab was included and cells were diluted (Fig. 8B). In contrast, almost half of the staining was lost in just 30 min under the same conditions without inclusion of anti-pMHC tetramer Ab (Fig. 8B). We also performed pMHC tetramer off rate experiments in the presence of anti–HLA-A2 Ab to prevent rebinding of TCRs (Fig. 8C) (12). These conditions exaggerate the dissociation of pMHC multimer from the cell surface and showed that addition of 2° Ab with the 1° Ab did not further alter the decay rate (Fig. 8C). The MFIs of staining in the presence of competing pMHC Ab highlighted the differences in staining intensities over time with the different conditions (Fig. 8D). We conclude that cross-linking of pMHC multimer substantially reduces its dissociation from the cell surface after staining. Presumably, this effect is also at play during regular staining and washing protocols. Such losses could be very substantial given that our own standard ICS protocol involves 12 washes and 3 incubation steps, thereby providing ample opportunity for pMHC multimer staining to decrease due to dissociation from the T cell surface. Overall, it appears that there is a large loss of tetramer from the cell surface over time when stained cells are incubated on ice as during most pMHC multimer, ICS, and Ab phenotyping experiments. This loss can be largely prevented by stabilizing pMHC multimer at the cell surface using anti-fluorochrome 1° Ab.

## Discussion

Fluorescent pMHC multimers are now part of the standard toolset for the study of Ag-specific T cells (1), but the binding affinity threshold for staining with these tools can be significantly higher than that required for T cell activation (2, 13). Thus, pMHC tetramers fail to stain all T cells that are capable of responding to the pMHC used in the multimer, and there is a pressing need for reagents that can stain T cells with relatively weak affinity TCRs such as those that predominate in cancer-specific, autoimmune, or MHC II–restricted T cell populations. In this study, we examined whether a combination of anti-fluorochrome unconjugated 1° Ab followed by anti-Ab conjugated 2° Ab could be used to boost the fluorescence signal during pMHC multimer staining and detection by flow cytometry. Our initial experiments with the ILA1 T cell clone and the weak 4L ligand showed that a combination of 1° and 2° Abs could boost the MFI by ∼20-fold compared with regular tetramer staining. A 6-fold boost in fluorescence was still observed when the staining was performed in the presence of the PKI dasatinib that we have previously shown affords considerable advantages during T cell staining with multimerized pMHC (1, 15, 18). Signal amplification by including a combination of 1° and 2° Abs was not wholly unexpected, although the 20-fold increase observed was higher than expected based on calculations of how much extra fluorochrome this procedure was expected to deliver.

We were also very surprised to observe that the majority of the signal boost apparent with a combination of 1° and 2° Ab was still present when only the unconjugated 1° Ab was used. The substantial increase in MFI observed in the presence of anti-pMHC tetramer Ab might represent an inexpensive and easy way to increase the utility of pMHC multimers and warranted further investigation. Recovery of ILA1 T cells spiked into HLA-A2^+^ PBMC using pMHC tetramers of the 4L, 5Y, and 8E variant that bind with *K*_D_s of 117, ∼250, and ∼2000 μM was 6, 0.1, and 0%, respectively, with regular tetramer staining. These levels increased to 100, 33, and 19%, respectively, when a 1° Ab was included during staining. Remarkably, all of the clone could be recovered with pMHC tetramers of all these ligands when 1° Ab was included with PKI and we were able to see effective staining of the ILA1 T cell with the 8E variant agonist. We conclude that the inclusion of anti-fluorochrome 1° Ab during pMHC tetramer staining substantially increases both the intensity of staining and the range of TCR–pMHC interactions that can be used to detect T cells using these reagents. The increase in staining we observed when an anti-pMHC multimer Ab is included is a general effect that was also seen with other Ab clones against PE and when allophycocyanin- or FITC-based pMHC multimers were used in combination with Abs against the relevant fluorochrome. Inclusion of 1° Ab during pMHC tetramer staining also enabled good detection of T cells even when substantially less pMHC reagent was used. The benefits of including Ab were evident when staining T cells specific for viral, tumor, and autoimmune Ags and with both pMHC tetramers and pMHC dextramers. There was a distinct hierarchy of cellular recovery of antitumor T cells from a PMBC sample that ran dextramer + 1° + 2° Abs > dextramer + 1° Ab > dextramer > tetramer + 1° + 2° Abs > tetramer + 1° Ab > tetramer. Thus, addition of Abs against pMHC multimers during staining improves MFI and cellular recovery with both pMHC tetramers and pMHC dextramers. The most sensitive staining protocol used a combination of: 1) pMHC dextramer; 2) PKI; 3) anti-fluorochrome unconjugated Ab; and 4) anti-Ab conjugated 2° Ab.

It is well documented that TCRs downregulate from the T cell surface once they are triggered (34). Thus, T cells that have recently engaged cognate Ag exhibit a lower surface density of TCR and are more difficult to stain with pMHC multimers (15). This issue becomes particularly problematic when attempting to identify self-specific T cells (anticancer or autoimmune) that tend to bear lower affinity TCRs (3) and might be expected to have had a reasonable chance of recent Ag encounter in vivo. When pMHC tetramer staining ELAGIGILTV-specific T cells in the TILs expanded from an HLA-A2^+^ patient with stage IV melanoma after exposure to autologous tumor, 80% of the original population could be recovered after pMHC tetramer staining with 1° and 2° Abs. This compared with just 29% with tetramer alone. Similarly, almost all of the 1E6 PPI-specific T cells spiked into HLA-A2^+^ PBMC after incubation with HLA-A2^+^ cells expressing PPI could be recovered using pMHC tetramer + 1° and 2° Abs, whereas none of the cells could be recovered when stained with pMHC tetramer alone. Thus, addition of Ab against pMHC multimers during cellular staining can considerably improve detection of self-specific T cells that have recently encountered Ag.

We finally examined the mechanism by which staining was enhanced. The rational of using an unconjugated 1° Ab in combination with a conjugated 2° Ab was to boost the amount of fluorochrome that could be loaded onto Ag-specific T cells using pMHC multimers. In some cases, this methodology increased the staining intensity of cognate T cells by ∼20-fold. Simple arithmetic suggested that the additional fluorochrome added with a combination of 1° and 2° Ab could not explain the majority of the increase in MFI we observed. Experiments confirmed that the majority of the increase in MFI observed with 1° and 2° Abs during staining as described could be induced by addition of just 1° Ab. Further experiments showed that the enhancement afforded by addition of Ab against pMHC tetramers extended to reagents manufactured with allophycocyanin and FITC specificities in addition to PE and could be induced with all Ab clones tested. These experiments, and enhancement observed when using anti-streptavidin Ab, rule out the possibility that our original observation was due to an Ab-induced effect on fluorochrome emission. Instead, it seemed more likely that the major effect observed was due to an increase in stabilization via a substantially reduced off rate. Experiments designed to look at tetramer off rates during standard staining incubations showed that there was a large loss of tetramer staining during the course of experiments in the absence of anti-pMHC tetramer Abs. Addition of an Ab against pMHC tetramer reversed the majority of this loss.

Although the inclusion of both unconjugated 1° and conjugated 2° Abs gave the best results, our laboratory now routinely stains using only the former Ab. Use of just 1° Ab provides the vast majority of the enhancement at very little cost (<$0.25 per stain). Addition of unconjugated 1° Ab does not introduce any risk of increased background staining that is possible with the further addition of fluorochrome-conjugated 2° Abs. Importantly, the procedures described in this study have been compatible with all of the polychromatic T cell phenotyping we have attempted to date, providing the tetramer + 1° ± 2° Abs are applied prior to other Abs. Nevertheless, it should be noted that anti-fluorochrome Abs are bivalent, resulting in the potential that if one binding site were not occupied by cross-linking pMHC multimer, then it could be available to bind Abs coupled to tandem dyes, leading to potential artifacts in phenotypic measurements. Although our own preferred staining protocol includes PKI staining and only 1° Ab, all of the methodologies used in this study show additive benefits for both the MFI of staining and the range of TCR interactions that are amenable to detection, thereby allowing researchers to adjust protocols to suit their own individual needs and circumstances.

In summary, we show that including Abs against pMHC tetramers or dextramers during cell staining can result in substantial improvements in both the MFI of staining and the range of TCR interactions amenable to detection, thereby revealing important cell populations that could not be identified otherwise. The best results were observed with a combination of pMHC multimer, PKI, anti-fluorochrome 1° Ab, and anti-Ab conjugated 2° Ab. Surprisingly, the majority of the benefits observed with this protocol were still evident when only the 1° Ab was included. In addition to increased MFI and a weaker TCR affinity threshold required for staining, inclusion of Ab also allowed use of log-fold lower pMHC multimer reagent concentrations. The mechanism for these unanticipated affects appears to involve stabilization of reagent capture at the T cell surface during the staining protocol. We anticipate that this improved methodology will become routinely adopted during pMHC multimer staining, as it represents a considerable improvement in the brightness of staining, an extension in the scope of interactions that can be detected, and large potential cost savings compared with existing technology.

## Supplementary Material

Data Supplement
