# Supplementary material for: Antibody Stabilization of Peptide–MHC Multimers Reveals Functional T Cells Bearing Extremely Low-Affinity TCRs
Source: J Immunol. 2014 Dec 1;194(1):463–74. doi: 10.4049/jimmunol.1401785 (PMC4273996; doi:10.4049/jimmunol.1401785)
Supplement: Data Supplement [file supp_194_1_463__index.html]

Antibody Stabilization of Peptide–MHC Multimers Reveals Functional T Cells Bearing Extremely Low-Affinity TCRs — Data Supplement 

# Antibody Stabilization of Peptide–MHC Multimers Reveals Functional T Cells Bearing Extremely Low-Affinity TCRs

## Data Supplement

**Files in this Data Supplement:**

- Supplemental Figure 1 (PDF)
