# Supplementary material for: Antibody Stabilization of Peptide–MHC Multimers Reveals Functional T Cells Bearing Extremely Low-Affinity TCRs
Source: J Immunol. 2014 Dec 1;194(1):463–74. doi: 10.4049/jimmunol.1401785 (PMC4273996; doi:10.4049/jimmunol.1401785)
Supplement: Data Supplement [file 1401785_JI_1401785_Supplemental_Figure_1.pdf]

Supplementary Figure 1. Tungatt et al.

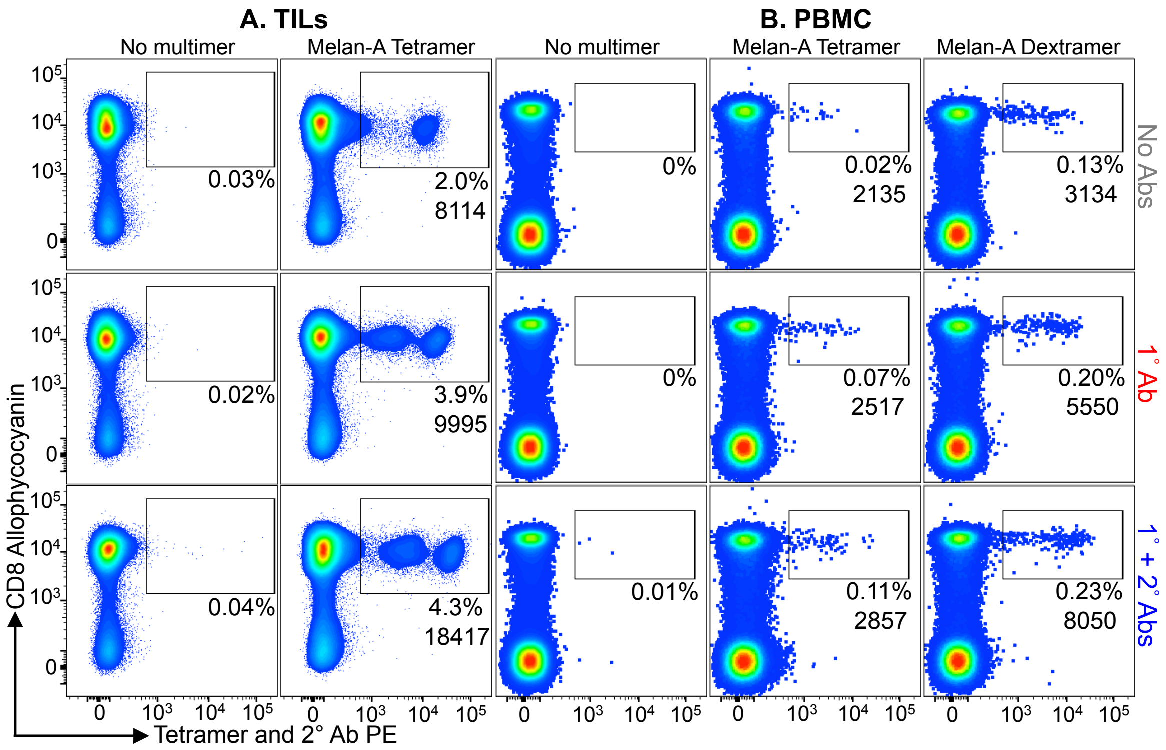

Supplementary Figure 1: Ex vivo staining and detection of T-cells is improved by the addition of an anti-fluorochrome and conjugated secondary antibody to standard pMHC multimer staining protocols. (A) TILs from a HLA-A2$^+$ metastatic melanoma patient were left unstained or stained with HLA-A2-ELAGIGILTV (from Melan-A) PE conjugated tetramers ± an anti-PE unconjugated 1° Ab ± a PE conjugated 2° Ab. These TILs did not stain with irrelevant tetramer (e.g. Figure 5). **(B)** Peripheral blood mononuclear cells (PBMCs) from a healthy donor were stained ex vivo with tetramers or dextramers (HLA-A2-ELAGIGILTV) and Abs as in A. Samples were minimally stained for viability, CD3 and CD8, with CD14 and CD19 also being stained in B. The tetramer$^+$ cells are expressed as a percentage of total cells (A) or CD8$^+$ cells (B) and the mean fluorescence intensity shown inset. Cells were pre-treated with PKI throughout.
